# Effects of predispersal insect seed predation on the early life history stages of a rare cold sand-desert legume

**DOI:** 10.1038/s41598-018-21487-7

**Published:** 2018-02-19

**Authors:** Yi J. Han, Jerry M. Baskin, Dun Y. Tan, Carol C. Baskin, Ming Y. Wu

**Affiliations:** 1 Xinjiang Key Laboratory of Grassland Resources and Ecology and Ministry of Education Key Laboratory for Western Arid Region Grassland Resources and Ecology, College of Grassland and Environment Sciences, Xinjiang Agricultural University, Ürümqi, 830052 China; 2 College of Biology and Environmental Sciences, Jishou University, Jishou, 416000 China; 3 Department of Biology, University of Kentucky, Lexington, KY 40506 USA; 4 Department of Plant and Soil Sciences, University of Kentucky, Lexington, KY 40506 USA

## Abstract

Seed predation by insects is common in seeds of Fabaceae (legume) species with physical dormancy (PY). However, the consequences of insect seed predation on the life history of legumes with PY have been little studied. In the largest genus of seed plants, *Astragalus* (Fabaceae), only one study has tested the effects of insect predation on germination, and none has tested it directly on seedling survival. Thus, we tested the effects of insect predation on seed germination and seedling growth and survival of *Astragalus lehmannianus*, a central Asian sand-desert endemic. Under laboratory conditions, seeds lightly predated in the natural habitat of this perennial legume germinated to a much higher percentage than intact seeds, and seedlings from predated and nonpredated seeds survived and grew about equally well. Further, in contrast to our prediction seedlings from predated seeds that germinated “out-of-season” under near-natural conditions in NW China survived over winter. The implication of our results is that individual plants from predated seeds that germinate early (in our case autumn) potentially have a fitness advantage over those from nonpredated seeds, which delay germination until spring of a subsequent year.

## Introduction

Predispersal seed predation by insects is common in members of the Fabaceae (legumes)^1–5^. However, many seed predation studies have not taken into account the possibility that partially-damaged seeds may be viable, germinable and capable of producing healthy plants. Thus, predated seeds typically have been lumped into the category of “seeds destroyed” without considering the different levels of predation that individual seeds experience in the field. It is obvious, then, that more attention needs to be given to the fate of predated seeds of legumes that are damaged but not killed by predation^6^.

According to the Nikolaeva-Baskin classification system of seed dormancy, there are five major kinds (classes) of seed dormancy. Dormancy in one of the classes, physical dormancy (PY), is caused only by a water-impermeable (“hard”) seed (or fruit) coat^7^. In nature, PY is broken by an abiotic environmental cue, such as high fluctuating temperatures, that cause a specialized structure in the seed (or fruit) coat, the “water gap,” to open, thereby allowing water to enter into and hydrate the seed, whereupon the seed can germinate over a wide range of temperatures in both light and dark^7^.

An artificial way to break dormancy in seeds with PY is to scarify them, i.e. breach the integrity of the water-impermeable seed (or fruit) coat by soaking the seeds in concentrated sulfuric acid (acid scarification) or by cutting a hole in the seed (or fruit) coat (mechanical scarification). PY is known to occur in species in 18 families of angiosperms (no gymnosperms)^7^. Of the 18 families that contain species with PY, seed predation has been studied most extensively in the Fabaceae. Given that breaching the seed coat breaks PY, it seems logical that a seed could be lightly predated (embryo remains viable) and be capable of germinating and growing into a reproductively mature plant, which in nature would depend on the tolerances and growth requirements of the seedling and perhaps on the season the seed germinates. Thus, if a predated seed germinates “out-of-season” (i.e. in a season prior to the one in which nonpredated seeds of the species typically germinate), the resulting plants may not survive until reproductive maturity, resulting in zero fitness for the early germinators^8,9^. If, on the other hand, predated seeds germinate earlier than is typical for nonpredated seeds of the species to do so and the resulting plants reached reproductive maturity, fitness might be higher for the early than that for the late germinators^10^.

Insect predation has been documented in seeds of many legumes^1–5^; however, the life history consequences of the predation have been documented in only a few legume species. Thus, the purpose of our research was to determine the effect of predispersal insect seed predation on the early life history stages (i.e. seed germination, seedling survival) of the rare cold sand-desert species *Astragalus lehmannianus* Bunge (Fabaceae) via both laboratory and field studies. Our hypotheses were that 1) lightly-predated seeds would imbibe water, germinate and produce healthy seedlings (potentially increase fitness), whereas heavily predated seeds would imbibe water but not germinate, or if seeds did germinate they would not produce healthy seedlings; and 2) predated seeds that germinate in near-natural conditions (experimental garden) “out-of-season” in autumn would not survive over winter (potentially decrease fitness). Taken together, these two hypotheses predict that insect predation is likely to have a negative effect on individual plant fitness and population growth rate (λ).

## Results

### Percentage of predated seeds in natural habitat

Seeds of *A*. *lehmannianus* are predated before dispersal, and the seed coat of mature seeds has holes of different sizes in them (Supplementary Figure S1). Mean seed predation in the four study populations ranged from 16.2 to 22.7%. Percentage of intact and of medium and high predated seeds did not differ significantly in the four study populations (Intact: *F*_3, 36_ = 85.30, *P* = 0.47; Medium: *F*_3, 36_ = 40.70, *P* = 0.75; High: *F*_3, 36_ = 63.90, *P* = 0.60). However, percentage of low predation was significantly higher in P2 than in the other three populations (*F*_3, 36_ = 85.30, *P* = 0.04, Table 1). In the same population, percentage of intact seeds was significantly higher than that of the three classes of predated seeds (P1: *F*_3, 36_ = 595.5, *P* < 0.001; P2: *F*_3, 36_ = 194.0, *P* < 0.001; P3: *F*_3, 36_ = 426.7, *P* < 0.001; P4: *F*_3, 36_ = 361.7, *P* < 0.001). However, there was no significant difference between percentages of low, medium and high predated seeds except in P1, where percentage of high predated seeds was significantly higher than that of medium predated seeds (Table 1).

**Table 1:** Percentage (mean ± se) of seeds damaged by insect predation in the four study populations of *Astragalus lehmannianus*. Different uppercase letters indicate significant differences within the same populations and different lowercase letters significant differences in the four populations (*P* < 0.05).

Population	Number of fruits	Total number of seeds	Percentage (%)			
			Intact	Low	Medium	High
P1	300	592	82.6 ± 2.1 ^Aa^	4.3 ± 1.0 ^BCb^	3.5 ± 1.4 ^Ca^	9.5 ± 1.6 ^Ba^
P2	300	595	77.2 ± 4.0 ^Aa^	10.3 ± 1.7 ^Ba^	5.5 ± 1.3 ^Ba^	6.9 ± 2.0 ^Ba^
P3	300	588	83.8 ± 2.9 ^Aa^	5.2 ± 1.6 ^Bb^	4.4 ± 1.1 ^Ba^	6.6 ± 1.6 ^Ba^
P4	300	588	81.5 ± 3.1 ^Aa^	5.8 ± 1.6 ^Bb^	4.9 ± 1.5 ^Ba^	7.8 ± 1.2 ^Ba^

### Effect of seed predation on imbibition and seed germination

#### Imbibition of water

**Figure 1:**
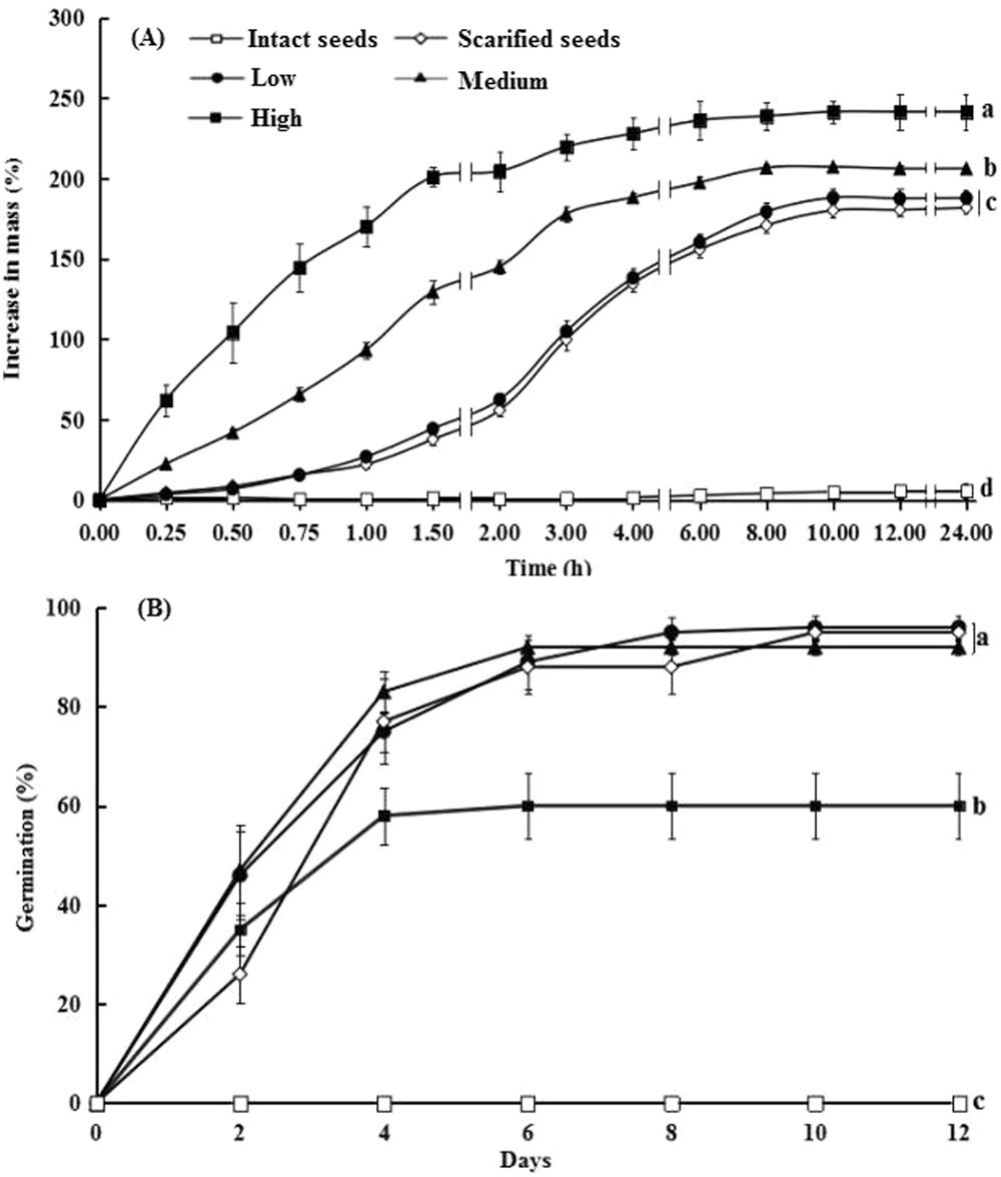
Time-course for increase in mass via water absorption at ambient laboratory conditions (**A**) and for germination (**B**) (mean % ± se) of intact nonscarified, scarified and of the three classes of seed predation of *Astragalus lehmannianus* in light at 30/15 °C. Different lowercase letters indicate significant differences for final increase in mass (**A**) and final germination percentage (**B**) (*P* < 0.05).

Seed predation had significant effects on increase in mass after imbibition (*F*_4, 15_ = 413.50, *P* < 0.001). Mechanically scarified and insect-predated seeds imbibed, whereas intact seeds did not (Fig. 1A). Scarified and low, medium and high predated seeds imbibed water rapidly in the first 3 hours and reached saturation after 8 to 10 h. The high predated seeds imbibed water more rapidly than the intact (*P* < 0.001), manually-scarified (*P* < 0.001) and low (*P* < 0.001) and medium predated seeds (*P* = 0.001), while the manually-scarified and low predated seeds (all *P* = 0.001) imbibed water more slowly than medium predated seeds.

#### Germination

**Figure 2:**
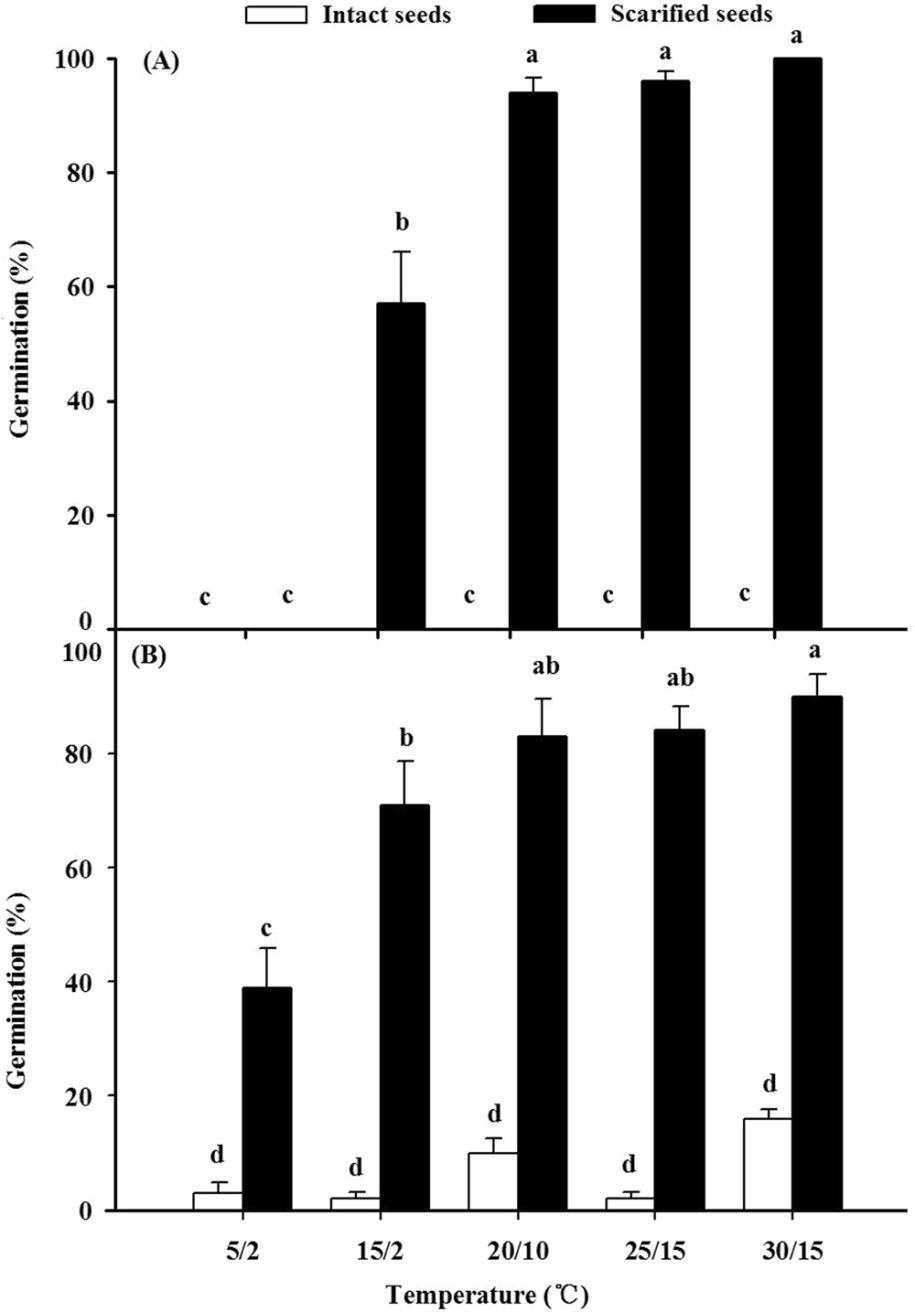
Germination (mean% + se) of fresh intact (▫) and scarified (▪) seeds of *Aslragalus lehmannianus* in light (**A**) and darkness (**B**). Bars in (**A**) and in (**B**) with different lowercase letters are significantly different (*P* < 0.05).

In the first germination experiment, intact seeds did not germinate at any of the five temperature regimes in light, while manually-scarified seeds germinated to ≥90% at all temperature regimes within 28d in light except at 5/2 °C and 15/2 °C (Fig. 2A). In darkness, a low percentage of the intact seeds germinated at all temperature regimes, while germination of scarified seeds ranged from 39% to 90% (Fig. 2B). A Three-way ANOVA showed that temperature, seed coat and light all had significant effects on seed germination via their main or interactive effects. However, the seed coat was the most important factor influencing seed germination as it alone and interactively explained 69.7% and 84.2%, respectively, of total sum of square in the simplified model (Supplementary Table S2).

In the second germination experiment, seed predation had significant effects on final germination percentage (*F*_4, 15_ = 122.73, *P* < 0.001). Intact seeds did not germinate at the optimum temperature for germination, while some manually-scarified seeds and those in the three classes of predation had germinated by day 2 and reached the highest germination percentage by day 7 (Fig. 1B). There was no significant difference between final germination (92–96%) of low-predated, medium-predated and manually-scarified seeds (all *P* > 0.05). However, only 60% of the heavily-damaged seeds germinated.

### Effect of seed predation on seedling survival and growth

Insect predation damaged cotyledons and radicles (Supplementary Figure S1). Low predation by insects damaged either the cotyledons or the radicle, whereas medium and/or high predation resulted in damage to cotyledons, radicle or both. Sixty-six percent of the seedlings from high predation had both cotyledons and radicles damaged (Supplementary Figure S2).

**Figure 3:**
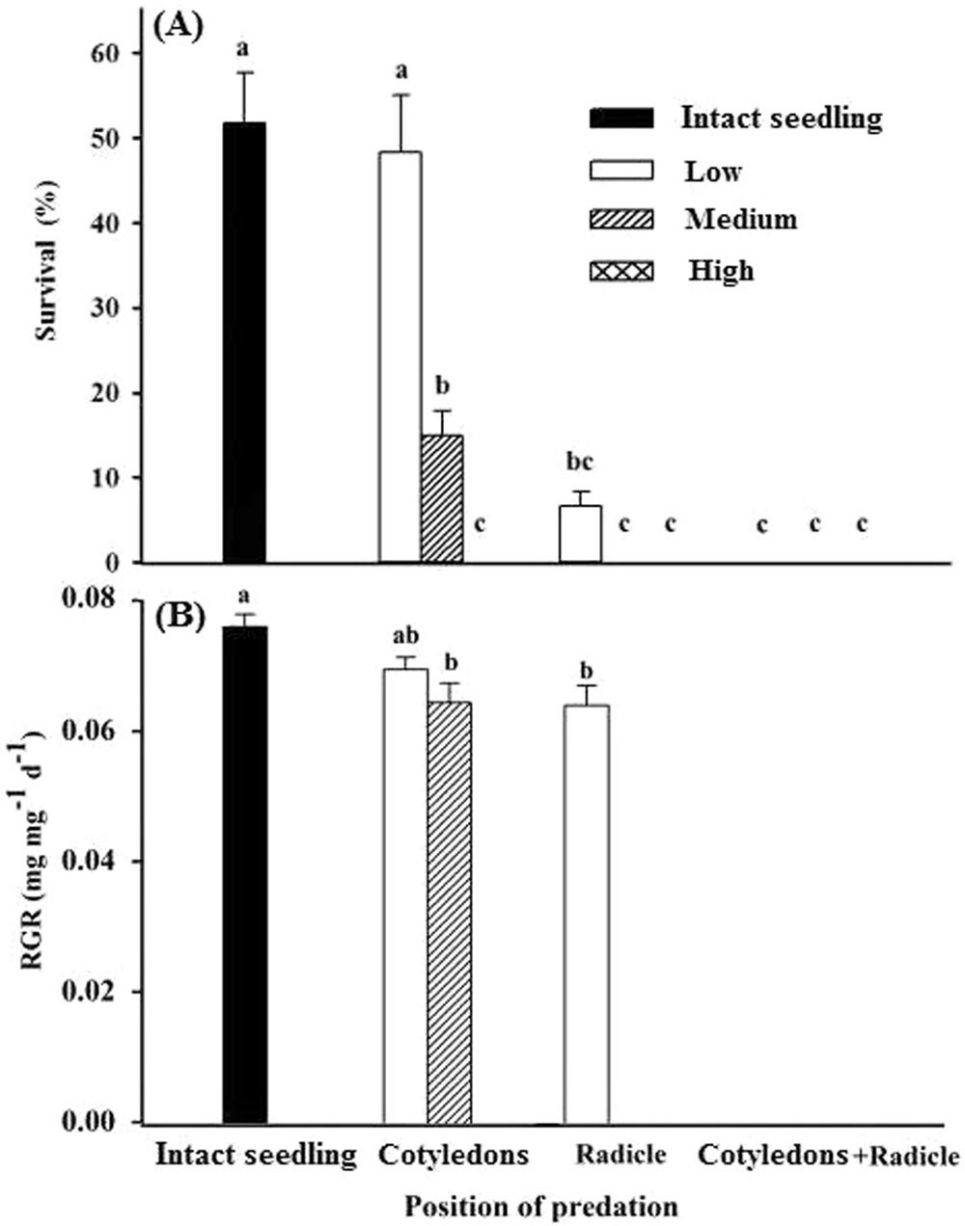
Effect of seed predation class on seedling survival (**A**) and relative growth rate (RGR) (**B**) (mean + se) in *Astragalus lehmannianus*. Intact seedlings germinated from scarified seeds, and thus neither the cotyledons nor radicle was damaged by predators. Bars with different lowercase letters are significantly different (*P* < 0.05).

Damage caused differences in growth and survival of the seedlings. At the end of the experiment, seedlings from intact seeds had significantly higher survival than those from seeds damaged by insects (all *P* < 0.001) except for seedlings from low-damaged cotyledons (*P* = 0.45). However, survival was 0% for seedlings from seeds with medium predation of radicle or of cotyledons + radicle and for seedlings from seeds with high predation of cotyledons, radicle or cotyledons + radicle (Fig. 3A). Predation position (*F*_2, 20_ = 15.56, *P* < 0.001), predation class (*F*_2, 20_ = 20.48, *P* < 0.001) and the interaction between them (*F*_4, 20_ = 11.32, *P* < 0.001) significantly influenced seedling survival.

RGR of seedlings from nonpredated seeds was significantly higher than that of seedlings from seeds with low-damaged radicles (*P* = 0.09) and medium-damaged cotyledons (*P* = 0.01), but not significantly higher than that of seedlings from seeds with low-damaged cotyledons (*P* = 0.28). RGR of seedlings from seeds with low- and medium-damaged cotyledons and those with low-damaged radicles did not differ significantly (Fig. 3B). Predation position (*F*_2, 90_ = 609.89, *P* < 0.001), predation class (*F*_2, 90_ = 606.54, *P* < 0.001) and the interaction between them (*F*_4, 90_ = 281.52, *P* < 0.001) had significant effects on seedling RGR and total biomass.

**Figure 4:**
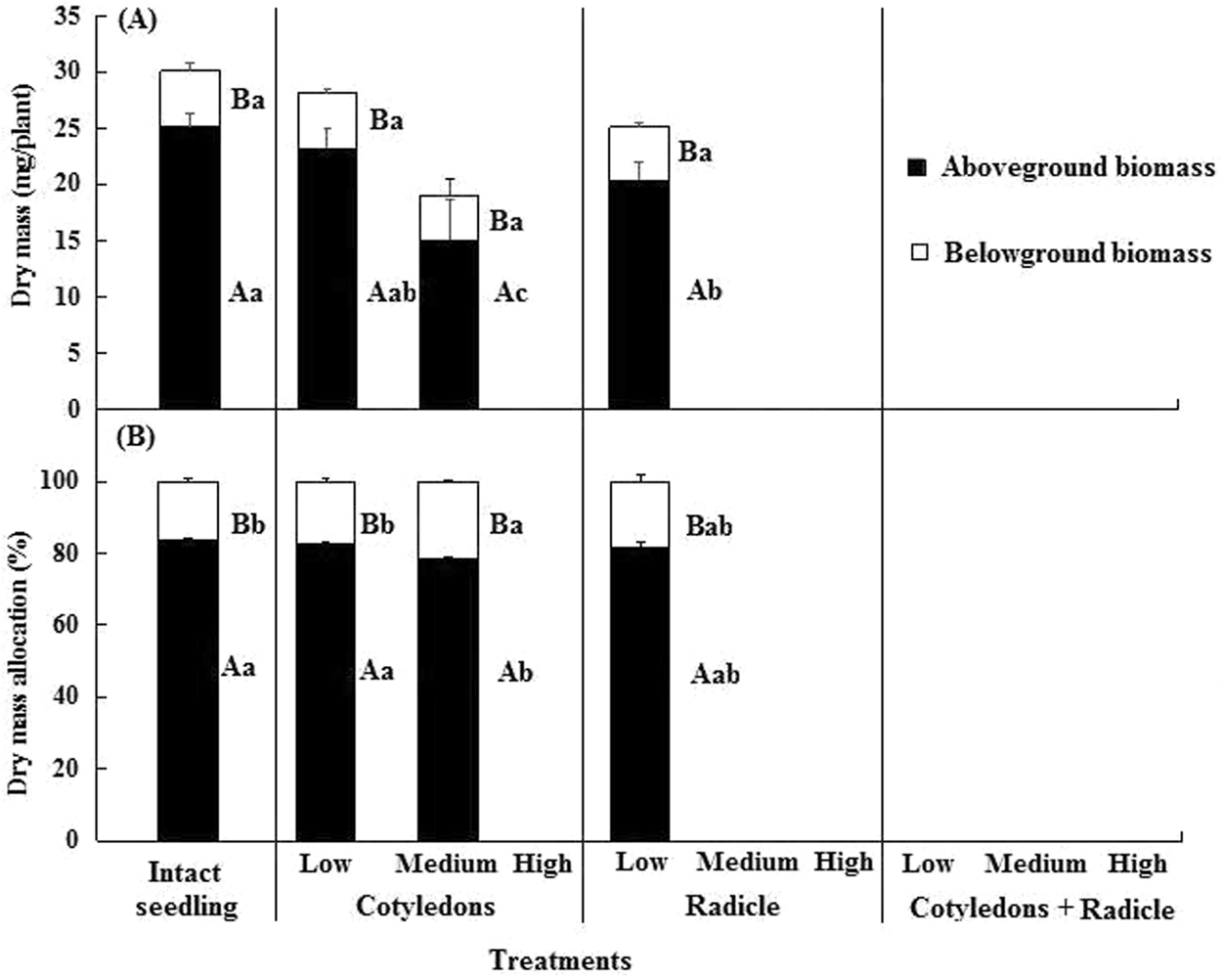
Effect of seed predation class on dry mass accumulation (**A**) and allocation (**B**) (mean + se) in *Astragalus lehmannianus* seedlings. Intact seedings germinated from scarified seeds and thus neither the cotyledons nor radicle was damaged by predators. In (**A**) and (**B**), different uppercase letters indicate significant differences between aboveground and belowground biomass in the same predation level and different lowercase letters significant differences in aboveground or belowground biomass among the predation classes (*P* < 0.05).

Dry mass of seedlings from seeds with low-damaged cotyledons (*P* = 0.79) and radicles (*P* = 0.10) was not significantly greater than that of seedlings from intact seeds, but it was significantly greater than that of seedlings from medium-damaged cotyledons (*P* < 0.001, Fig. 4A). Seedlings from intact seeds and those from seeds in the three damage classes allocated more biomass to aboveground than to belowground growth. There was no significant difference between the proportion of aboveground and belowground biomass of seedlings from intact seeds and that of seedlings in different classes of insect damage to the cotyledons and radicle (Fig. 4B). Thus, seedlings from predated seeds that survived grew as well, or almost as well, as those from nonpredated seeds.

### Effect of predation and watering on seed germination and seedling survival in experimental garden

**Figure 5:**
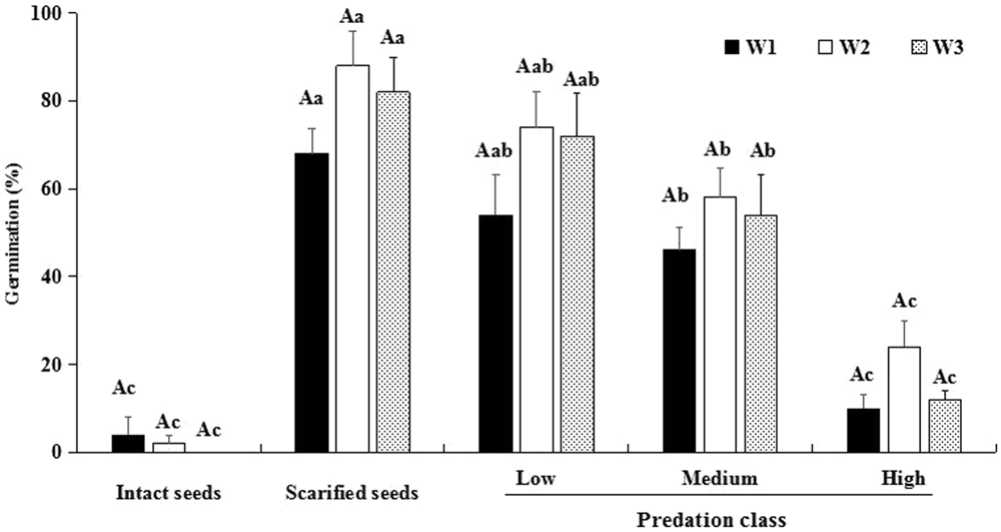
Germination (mean % + se) of intact, scarified and of the three classes of seed predation of *Astragalus lehmannianus* under different water conditions. W1, natural precipitation; W2, watered only until germination occurred; W3, watered until 1 September 2016. Same uppercase letters indicate no significant differences between watering regime in the same seed treatment (*P* > 0.05), and different lowercase letters indicate significant differences between seed treatments in the same watering regime across the five treatments.(*P* < 0.05).

Within the same watering condition, there was no significant difference between germination of scarified seeds and that of low-predated seeds (W1: *F*_4, 20_ = 22.87, *P* = 0.11; W2: *F*_4, 20_ = 29.56, *P* = 0.15; W3: *F*_4, 20_ = 25.82, *P* = 0.33). However, germination of intact and of medium- and high-predated seeds was significantly lower than that of scarified and low-predated seeds (all *P* < 0.05; Fig. 5). In the same predation class, germination under natural precipitation and watering treatments did not differ significantly (Intact seeds: *F*_2, 12_ = 0.67, *P* = 0.56; Scarified: *F*_2, 12_ = 1.95, *P* = 0.19; Low: *F*_2, 12_ = 1.48, *P* = 0.27; Medium: *F*_2, 12_ = 0.72, *P* = 0.51; High: *F*_2, 12_ = 3.00, *P* = 0.08). Although a proportion of intact, scarified and the three predation classes of seeds germinated, no seedlings survived under natural precipitation or in the no watering after germination treatment (Table 2). However, >50% of seedlings from scarified and low and medium predated seeds watered every 3 days grew into juveniles and survived over winter. Survival of seedlings from high predated seeds differed from that of seedlings from scarified and low predated seeds but not from that of seedlings from medium predated seeds. Only a very low percentage of the intact seeds germinated, and 96.6% of them were still viable after burial in soil for 1 year.

**Table 2:** Percentage (mean ± se) before and after overwintering survival of seedlings from scarified and the three predation classes of *Astragalus lehmannianus* seeds. Different superscript letters within a column indicate significant differences (*P* < 0.05). Only one nonscarified seed germinated (in natural precipitation), and the seedling died soon after germination.

Seedling source	Percentage surviving before overwintering (%)			Percentage surviving after overwintering (%)		
	Natural precipitation	No water after germination	Watered every 3 days	Natural precipitation	No water after germination	Watered every 3 days
**Scarified seeds**	0	0	84.6 ± 4.1 ^a^	0	0	79.3 ± 3.7 ^a^
**Low**	0	0	62.8 ± 11.6 ^ab^	0	0	73.3 ± 19.4 ^a^
**Medium**	0	0	50.6 ± 8.5 ^ab^	0	0	50.0 ± 22.3 ^ab^
**High**	0	0	30.0 ± 20.0 ^b^	0	0	10.0 ± 10.0 ^b^

## Discussion

In general, it seems that plants of *A*. *lehmannianus* produced from seeds with more than about 5–15% predation will almost invariably have zero fitness (i.e. die before producing seeds), even under good conditions for growth and survival. Survival of seedlings with <5% damage to radicle (and 0% to cotyledons) was only about 5% under laboratory conditions. In which case, no plants of *A*. *lehmannianus* produced from seeds with ≥5% predation on the radicle are expected to survive in the cold desert. Thus, our first hypothesis that lightly-predated seeds would germinate and produce healthy seedlings, whereas heavily predated seeds would not germinate or produce healthy seedlings was supported. For *Astragalus australis* var. *olympicus*, an alpine endemic of the Pacific Northwest (USA), <5% tissue removal by a weevil seed predator scarified the seeds (broke PY). Ninety-two and five-tenths percent of the seeds were viable, and they germinated and produced healthy seedlings in a germinator. However, removal of >5% of the tissue killed the seeds^11^. Nothing was said about seedling survival.

**Figure 6:**
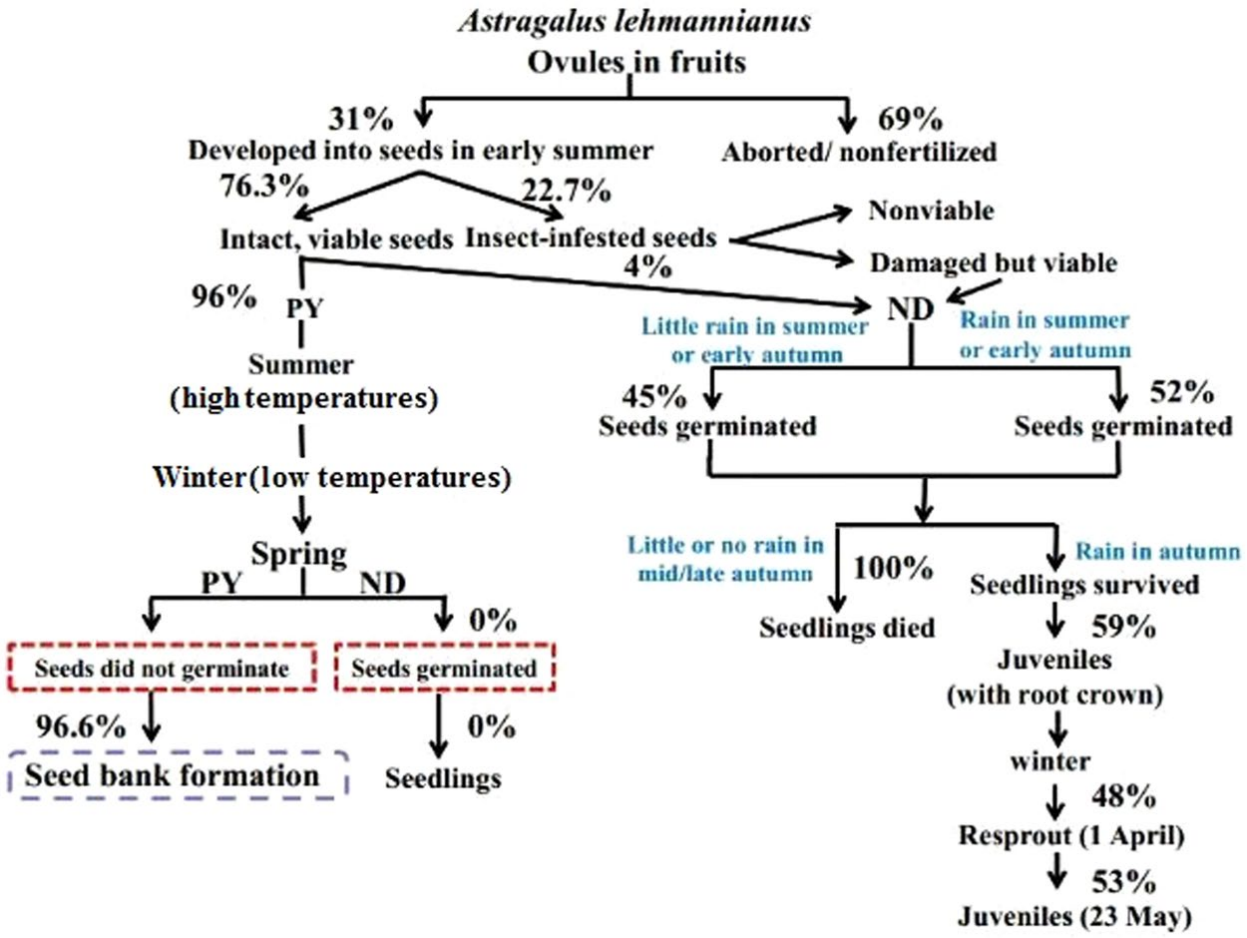
Conceptual model of the effects of “out-of-season” germination due to seed predation on the early life history stages of *Astragalus lehmannianus* in the field. ND, seeds nondormant; PY, physical dormancy.

*Astragalus* is the largest genus of seed plants (c. 2500 species)^12^. Yet, although predispersal seed predation by insects has been documented in at least 16 species of *Astragalus* (Supplementary Table S3), we are aware of only one study on *Astragalus* that measured the effects of seed predation by insects at the individual/population level. In the study by Combs *et al*.^13^, seed loss via insect predation on the rare species *A*. *sinuatus* negatively affected individual plant fitness (i.e. reduced the number of seeds produced per plant). However, the seed loss did not translate directly into demographic decline as shown by a seed addition experiment, which did not result in an increase in establishment rates of seedlings and subsequent juveniles in populations of *A*. *sinuatus*. Further, only one study other than ours has shown that a portion of the predated seeds of an *Astragalus* species remain viable and can germinate^11^. Kaye^11^ presented a “flow diagram” that included the fate of viable, predated seeds, but he did not attempt to determine what happened to them in the field. Thus, our study is the first one to measure the effects of a seed predator on seedling growth and survival in an *Astragalus* species under field conditions (experimental garden), and the results are summarized by a conceptual model (Fig. 6). The results are of particular significance in showing, to our surprise, that seedlings from predated seeds of *A*. *lehmannianus* that germinate “out-of-season” (in autumn) in the cold sand-desert climate can survive the winter if there is sufficient rainfall in autumn (in our study by hand-watering) for seeds to germinate and for seedlings to become established. Thus our second hypothesis that predated seeds that germinate in near-natural conditions (experimental garden) “out-of-season” in autumn would not survive over winter was not supported. In contrast, seeds that are not scarified by insect predation do not germinate in their first year following dispersal. These results suggest that insect predation has the potential to increase fitness of individual plants, i.e. by causing predated seeds to germinate earlier than nonpredated seeds.

A few studies on seed predation in legumes other than *Astragalus* species have measured growth and survival of seedlings from viable predated seeds. Predation by tortricid moths broke PY of *Vicia sativa* seeds, and in the field they germinated in autumn. However, seedlings did not survive the winter. Only plants from nonpredated seeds, which germinated in spring, survived to seed maturity^8,9^. Light-, medium- and heavily-infested seeds of *Lotus corniculatus* germinated and produced healthy plants^6^. Mass of juveniles grown from damaged seeds did not differ from those grown from nondamaged seeds. Survivorship of juveniles over winter was good. In general, seedlings from *Acacia greggii* and *Parkinsonia florida* seeds infested with one bruchid larva grew less well than those from control and mechanically scarified seeds, but the impact was relatively more negative for *P*. *florida* than for *A*. *greggii*. In both species, height and aboveground and belowground biomass declined as number of larva per seed increased from one to three. Seed germination of *Lathyrus japonicus* was promoted in autumn by a bruchid beetle, and seedling survival in the field was higher than that of nonpredated seeds, which germinated the subsequent summer^10^.

With regard to possible fitness effects of predation by insects on seeds of *Vicia*, *Lotus* and *Lathyrus*, the results are mixed. Seedlings of *Vicia sativa* from predated seeds germinated in autumn, and seedlings did not survive the winter^8,9^. On the other hand, nonpredated seeds germinated in spring (typical germination season), and the resulting plants produced seeds. These results strongly imply that dormancy break by an insect seed predator that causes seeds of this species to germinate “out-of-season” negatively affects fitness. Juveniles of *Lotus corniculatus* had good survivorship when subsequently allowed to overwinter outdoors^6^. The much earlier germination of weevil-infested seeds than of nonpredated seeds suggests that insect predation may increase λ of this species. Survival of *Lathyrus japonica* seedlings from predated seeds that germinated in autumn was higher than that of seedlings from nonpredated seeds that germinated the subsequent summer^10^. These results suggest that the seed-feeding bruchid *Bruchus loti* might have a positive effect on λ and on individual plant fitness.

Although insect seed predators kill many legume seeds with PY, they may break PY in a portion of the seeds by scarifying the water-impermeable (“hard”) seed coat without killing the embryo. Dormancy break in a portion of the seed crop of legumes by insect predation does not seem to be uncommon in species whose seeds are predated by insects. Heavily-predated seeds may not germinate, and if they do the seedlings are not likely to be healthy. Seedlings from lightly-predated seeds are likely to survive and grow well under laboratory and greenhouse conditions. In some cases, but not all, seedlings/juveniles from insect-predated seeds that germinate “out-of-season” survive under natural field conditions. However, we suggest that insect seed predation may, or may not, be demographically advantageous to species whose seeds have PY, including *Astragalus lehmannianus*, depending on year-to-year differences in seasonal rainfall, which may be affected by climate change.

This study on predispersal seed predation in *Astragalus lehmannianus* is only the first of many steps required to determine to what extent, if any, the seed predator affects the population biology of this rare cold-desert endemic. Our prediction is that the insect seed predator has little or no effect on overall population growth rate (λ) of this species and thus that Δλ due to predispersal insect seed predation is 0 or only slightly negative. We further predict that λ in *A*. *lehamnnianus* is not pollinator (pollen), seed or microsite limited.

## Materials and Methods

### Study area and seed collection

Freshly-matured seeds of *Astragalus lehmannianus* were collected from 10 individuals in each of four natural populations growing in the Gurbantunggut Desert, a sand-desert of the Junggar Basin of Xinjiang Province, China, in July 2015 and from the same three populations plus an addition one in July 2016 (Supplementary Table S1). In the laboratory, seeds were separated from the fruits (pods) and stored in paper bags at room temperature and humidity (18–30 °C, 20–30% RH) until used.

The Junggar Basin (N:44°15′—46°50′, E: 84°50′—91°20′) has a temperate continental climate. Mean annual temperature (1982 to 2012) is 5.8 °C, mean temperature of the coldest (January) and hottest (July) months −16.1 °C and 23.4 °C, respectively, and mean annual precipitation (including rain and snow) 180 mm (Data from China Meteorological Data Service Center). Annual potential evaporation is >2000 mm^14^.

### Study system

*Astragalus lehmannianus* (Fabaceae, subfamily Papilionoideae) is an upright (to at least 70 cm in height), nonclonal perennial herb that produces a taproot >0.5 m in vertical length (sand depth). It occurs in Russia, Turkmenistan, Kazakhstan, Uzbekistan and China^15^. In China, the species is distributed only in stationary and semi-stationary dunes in the Gurbantunggut Desert in Xinjiang Province, northwest, China (Supplementary Figure S3). Vegetative shoot buds present on the root crown elongate in April, and yellow, papilionaceous flowers appear in late May to early June. The ovary contains 6–8 ovules arranged linearly at the base of the style. *A*. *lehmannianus* flowers exhibit a mixed-mating system with cross pollination achieved only by *Zebramegilla albigena* (Hymenoptera, Apidae), and pollination is via the “valve” mechanism^16^. Thus, when the insect forces the wing-keel complex downwards the reproductive column emerges from the keel, and a little cloud of pollen is released. After *Z*. *albigena* visits a flower, the flower parts return to their initial position, and thus flowers can be visited several times. Fruits mature in late-June to early-July, and our field observations showed that seed set was low. Ripe pods contain only 1–2 mature seeds, and the other 4–7 ovules are aborted or not fertilized. Altogether, 69% of the ovules were aborted and/or not fertilized, and 31% of them matured into seeds in 2016, the year of our study. Of the 31% of the ovules that matured into seeds, 76.3% of them were intact (nondamaged), and 22.7% were damaged to various degrees by larvae of an insect seed predator, which we have identified as *Etiella zinckenella* Treitschke (Lepidoptera, Pyralididae)^17^ (Supplementary Figure S3). Females of *E*. *zinckenella* generally lay eggs on young fruits of *A*. *lehmannianus*, and the larvae bore into the pods and damage immature seeds. The synsepalous calyx is papery and shaped like a cocoon. As seeds mature, the calyx increases in size and encloses the mature fruit when it is dispersed. After dispersal, seeds are released from the pods/calyx on the sand surface (Supplementary Figure S3).

### Percentage of predated seeds in natural habitat

To determine the percentage of predated, nonaborted mature seeds, we haphazardly chose 10 plants from each of four study populations in July 2016, when fruits had matured completely. Thirty pods were chosen haphazardly from each plant, and the seeds were divided into nondamaged and three damaged categories. Photographs of seeds, embryos and seedlings for each category were taken using a calibrated Nikon SMZ1000 light microscope and imported into Motic Images Plus 2.0 software to measure the area of predation on them. Based on the area of tissue removed, the predated seeds were classified as low predation (<5%), medium predation (15–25%) and high predation (30–50%) (Supplementary Figure S1). The percentage of each predation class was calculated as (N_i_/N_t_) × 100, where N_i_ is the number of seeds from a predation class and N_t_ number of intact+predated seeds. The total percentage of predated seeds is the sum of the percentage of seeds from the three predation classes.

### Effect of seed predation on imbibition and seed germination

All studies on the effects of seed predation on imbibition, germination and seedling growth and survival were conducted using a composite of seeds from the four study populations. That is, seeds from the four study populations were pooled and then sorted into the five treatment categories. Fresh seeds collected on 1 July 2016 were used in experiments within 1 week.

#### Imbibition of water

To determine if predated seeds are permeable to water, imbibition was compared in intact, manually-scarified and the three classes of predated seeds. Seeds were scarified individually with a single-edge razor blade (mechanical scarification), and four replicates of 25 scarified, nonscarified and the three classes of predated seeds each were tested. Each replicate of treated and nontreated seeds was weighed using a Sartorius BS210S electronic balance (0.0001 g) and placed on filter paper moistened with distilled water in Petri dishes in the laboratory. At time zero and at 15-min intervals for the first hour, and after 1.5, 2, 3, 4, 6, 8, 10, 12 and 24 h seeds were removed from the filter paper, blotted dry and weighed. Percentage increase in seed mass was calculated using the following equation: % increase in mass = [(Wi-Wd)/Wd] × 100, where Wi = seed mass after each of various times of imbibition and Wd = mass of fresh seed at time zero.

#### Germination

The purpose of this experiment was to compare germination percentages of intact seeds, manually-scarified seeds and the three classes of predation. There were two germination experiments. In the first experiment, intact and manually-scarified seeds were incubated at daily (12/12 h) temperature regimes of 5/2, 15/2, 20/10, 25/15 and 30/15 °C in light (12 h of ≈100 μmol m^−2^s^−1^, 400–700 nm, cool white fluorescent light each day) and constant darkness (Petri dishes placed in light proof black bags) for 28d. In the second experiment, intact (control) and manually-scarified seeds and seeds with low predation, medium predation and high predation were incubated at optimum conditions for germination (30/15 °C, light) for 28d.

In both experiments, seeds were incubated in 9-cm-diameter plastic Petri dishes on two layers of Whatman No. 1 filter paper moistened with distilled water, and four replicates of 25 seeds each were used for each treatment. Germination in light was monitored daily for 28d and germinated seeds counted and removed from the Petri dishes. Seeds incubated in darkness in the first experiment were checked for germination only at the end of the experiment.

### Effect of seed predation on seedling survival and growth

The purpose of this experiment was to compare growth and survival of seedlings from intact seeds and from seeds in the three classes of predation. On 5 April 2016, 150 intact seeds and 1000 predated seeds collected in July 2015 were placed in 9-cm-diameter Petri dishes on two layers of Whatman No.1 filter paper moistened with distilled water (25 seeds per dish) and incubated in light at 30/15 °C. Intact seeds were manually- scarified by removing about 5% of the seed coat without injuring the underlying cotyledons. Based on the part of the predated seed that was damaged, seedlings were divided into three categories (cotyledons, radicle and cotyledons + radicle) with three damage classes (i.e. low, medium and high) for each category (Supplementary Figure S1). Five-hundred and forty damaged seedlings (20 seedlings per pot × 3 damaged positions × 3 damage classes × 3 pots) and 60 seedlings from nonpredated seeds (20 seedlings per pot × 3 pots) were transplanted into pots (18 cm depth and 25 cm diameter) filled with sand from the natural habitat of *A*. *lehmannianus* on 8 April 2016. The seedlings were grown in the laboratory (20–25 °C) in diffuse natural light plus fluorescent room light. The sand was watered daily throughout the experiment. Seedling survival was recorded daily until the fourth true leaf of seedlings from intact seeds was produced (i.e. 17 May 2016). At the beginning and end of the experiment, 10 seedlings were haphazardly chosen from each treatment, carefully cleaned of sand and separated into aboveground and belowground biomass. Plant parts were dried at 80 °C for 48 h and weighed using the Sartorius electronic balance. Seedling relative growth rate (RGR) was calculated: RGR = (ln W_2_ − lnW_1_)/t_2_ − t_1_, where W_1_ and W_2_ is the dry mass of a seedling at the beginning (W_1_) and end (W_2_) of the experiment, and t_2_ − t_1_, is the time (days) between measurements.

### Effect of predation and watering on seed germination and seedling survival in experimental garden

The purpose of this experiment was to compare germination and survival of seedlings from predated and intact seeds of *A*. *lehmannianus* in an experimental garden under hand-watered (no natural precipitation during the growing season, i.e., plants covered with clear plastic during natural rainfall events) and natural (only natural precipitation) soil moisture conditions. One-hundred and fifty haphazardly-chosen intact, manually-scarified and low, medium and high predated seeds each collected on 1 July 2016 were sown on 15 July 2016 in plastic pots 18 cm deep and 21 cm in diameter (with drainage holes in the bottom) filled with sand from the natural habitat of *A*. *lehmannianus*. The pots were divided equally into three watering treatments: (1) no water, received only natural rainfall throughout experiment (W1); (2) watered only until germination occurred (W2); and (3) watered until 1 September 2016 (W3). After 1 September 2016, all pots received only natural precipitation. There were five replications of 10 seeds per pot for each treatment [5 seed treatments × 3 watering regimes × 5 replications = 75 pots with 10 seeds each]. The experiment was carried out in the experimental garden on the campus of Xinjiang Agricultural University, Urümqi, China, located at the southern edge of the Junggar Basin. In the watered treatments, the sand was watered to field capacity every 3d. Newly-germinated seedlings were marked with toothpicks. Germination and seedling survival were monitored at 3-d intervals from the time of sowing in July 2016 until 1 September 2016. Then, the pots were checked on 1 April and 23 May (spring) to determine survival of seedlings/juveniles from seeds that germinated “out-of-season” (i.e. in autumn). Finally, three pots of intact seeds were randomly chosen (1 pot × 3 treatments = 3 pots) on 29 June 2017 and the sand sieved to determine the number of seeds still viable after 1 year of burial.

### Data analysis

To determine significant differences in percentage of intact and damaged seeds (i.e., low-, medium- and high predated seeds) in the four natural study populations and in the same population, we used one-way ANOVA.

To understand the effects of seed predation on imbibition and seed germination, we used one-way ANOVA to determine significant differences in increase in mass and final germination percentage among the treatments (i.e. intact, scarified and the three seed predation classes) for the “Imbibition of water” experiment and “the second germination experiment”. In the first germination experiment, three-way ANOVA was used to test the effects of light, temperature and seed coat treatment (i.e. intact vs. scarified) and their interactions on germination. In addition, we simplified the full model by removing some of the main and interactive effects by using the AIC criteria, if AIC values of the simplified model decreased or at least did not increase^18^. Based on the simplified model, we also calculated the relative importance of each term on seed germination as the percent of total sum of square(SS%) that each term explained^19^.

In order to determine the effect of seed predation on seedling survival and growth, **t**he proportion of the three positions (cotyledons, radicle and cotyledons + radicle) damaged in each of the three classes (i.e. low-, medium- and high predated seeds) were tested by one-way ANOVA. Two-way ANOVA was used to test for significant (P < 0.05) main effects (predation class and predation position) and their interactions on seedling survival, RGR (relative growth rate) and total biomass of seedlings. Independent-sample T-tests were used to compare differences between aboveground and belowground seedling biomass and allocation in the same predation classes.

In the “Effect of predation and watering on seed germination and seedling survival in experimental garden” experiment, one-way ANOVA was used to test the differences in germination between watering regimes in the same predation class and between seed treatments (i.e. intact, scarified and the three seed predation classes) in the same watering regime. In the same way, the percentage before and after overwintering survival of seedlings from scarified and the three predation classes of seeds under different watering regimes were tested.

If ANOVA showed significant differences, Tukey’s HSD test was performed for multiple comparisons to determine significant differences among treatments. All data analyses were performed with SPSS 19.0 software (SPSS Inc., Chicago, Illinois, USA) except for the second germination experiment, which was conducted in R environment^20^ to simplify model using AIC criteria.

## Electronic supplementary material

Supplementary Figure S1

Supplementary Figure S2.

Supplementary Figure S3.

Supplementary Table S1.

Supplementary Table S3.

Supplementary Table S2.
