# Supplementary material for: Effects of predispersal insect seed predation on the early life history stages of a rare cold sand-desert legume
Source: Sci Rep. 2018 Feb 19;8:3240. doi: 10.1038/s41598-018-21487-7 (PMC5818501; doi:10.1038/s41598-018-21487-7)

Effects of predispersal insect seed predation on the early life history stages of a rare cold sand-desert legume

Yi J. Han[1], Jerry M. Baskin[1, 3] , Dun Y. Tan[1,2*], Carol C. Baskin[1, 3, 4] and Ming Y. Wu[1]

[1] Xinjiang Key Laboratory of Grassland Resources and Ecology and Ministry of Education Key Laboratory for Western Arid Region Grassland Resources and Ecology, College of Grassland and Environment Sciences, Xinjiang Agricultural University, Ürümqi 830052, China

[2] College of Biology and Environmental Sciences, Jishou University, Jishou 416000, China

[3] Department of Biology, University of Kentucky, Lexington, KY 40506, USA.

[4] Department of Plant and Soil Sciences, University of Kentucky, Lexington, KY 40506, USA.

*Correspondence and requests for materials should be addressed to D.Y.T.

(tandunyan@163.com)

Supplementary Figure S2. Proportion (mean + se) of cotyledons, radicle and cotyledons + radicle of embryos of *Astragalus lehmannianus* damaged in three classes of predation. Bars with different lowercase letters are significantly different between cotyledons, radicle and cotyledons + radicle across the three predation levels ($P<0.05$).

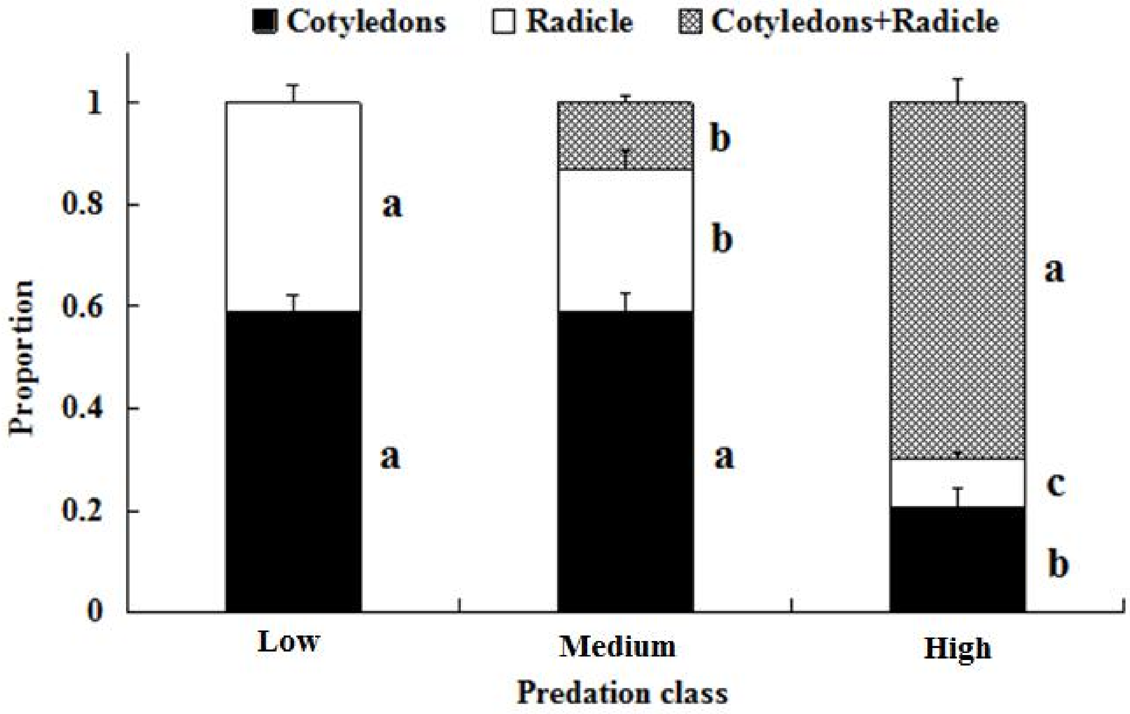

Supplement: Supplementary file 2 — Supplementary Figure S2. [file 41598_2018_21487_MOESM2_ESM.pdf]
