# Supplementary material for: Effects of predispersal insect seed predation on the early life history stages of a rare cold sand-desert legume
Source: Sci Rep. 2018 Feb 19;8:3240. doi: 10.1038/s41598-018-21487-7 (PMC5818501; doi:10.1038/s41598-018-21487-7)

Effects of predispersal insect seed predation on the early life history stages of a rare cold sand-desert legume

Yi J. Han[1], Jerry M. Baskin[1, 3] , Dun Y. Tan[1,2*], Carol C. Baskin[1, 3, 4] and Ming Y. Wu[1]

[1] Xinjiang Key Laboratory of Grassland Resources and Ecology and Ministry of Education Key Laboratory for Western Arid Region Grassland Resources and Ecology, College of Grassland and Environment Sciences, Xinjiang Agricultural University, Ürümqi 830052, China

[2] College of Biology and Environmental Sciences, Jishou University, Jishou 416000, China

[3]Department of Biology, University of Kentucky, Lexington, KY 40506, USA.

[4]Department of Plant and Soil Sciences, University of Kentucky, Lexington, KY 40506, USA.

*Correspondence and requests for materials should be addressed to D.Y.T.

(tandunyan@163.com)

Supplementary Figure S3. Individual plant (A); flowers nearly enclosed by calyx (B); immature fruits, each enclosed by an inflated calyx (C); longitudinal section of mature fruit with seeds inside (calyx has been removed) (D); larva (arrow) of *Etiella zinckenella* (E).

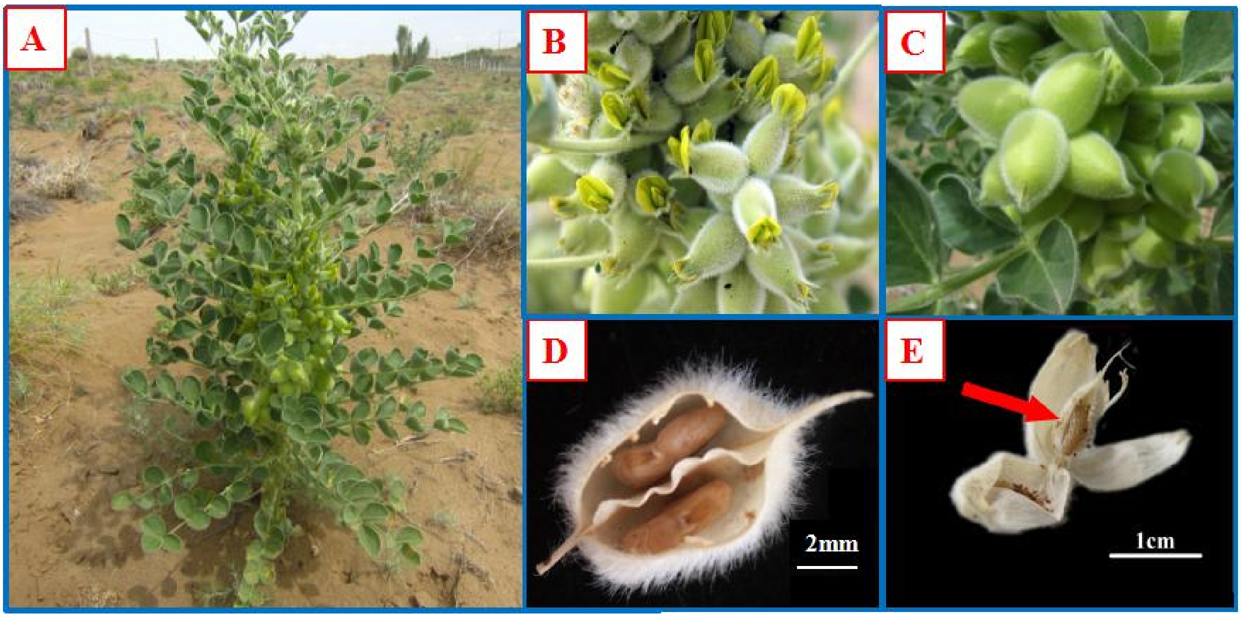

Supplement: Supplementary file 3 — Supplementary Figure S3. [file 41598_2018_21487_MOESM3_ESM.pdf]
