# Supplementary material for: Effects of predispersal insect seed predation on the early life history stages of a rare cold sand-desert legume
Source: Sci Rep. 2018 Feb 19;8:3240. doi: 10.1038/s41598-018-21487-7 (PMC5818501; doi:10.1038/s41598-018-21487-7)
Supplement: Supplementary file 4 — Supplementary Table S1. [file 41598_2018_21487_MOESM4_ESM.pdf]

Effects of predispersal insect seed predation on the early life history stages of a rare cold sand-desert legume

Yi J. Han[1], Jerry M. Baskin[1, 3] , Dun Y. Tan[1,2*], Carol C. Baskin[1, 3, 4] and Ming Y. Wu[1]

[1] Xinjiang Key Laboratory of Grassland Resources and Ecology and Ministry of Education Key Laboratory for Western Arid Region Grassland Resources and Ecology, College of Grassland and Environment Sciences, Xinjiang Agricultural University, Ürümqi 830052, China

[2] College of Biology and Environmental Sciences, Jishou University, Jishou 416000, China

[3]Department of Biology, University of Kentucky, Lexington, KY 40506, USA.

[4]Department of Plant and Soil Sciences, University of Kentucky, Lexington, KY 40506, USA.

*Correspondence and requests for materials should be addressed to D.Y.T.

(tandunyan@163.com)

Supplementary Table S1. Geographical location and number of plants (flowering and nonflowering, but not including seedlings) in the four study populations of *Astragalus lehmannianus* in Xinjiang Province, China, in 2016.

Population	Site	Longitude	Latitude	Elevation	Total number of plants
**P1**	Wutong valley, north of Fukang city	87°53'09"**E**	44°21'57"**N**	447m	＞200
**P2**	Cainan of Fukang City	88°08'28"**E**	44°22'16"**N**	507m	42
**P3**	National Defense Road No. 216, Jimsar	88°47'13"**E**	44°23'55"**N**	537m	47
**P4**	Provincial Road No. 240, Qitai	89°37'36"**E**	44°07'51"**N**	674m	35
