# Supplementary material for: Effects of predispersal insect seed predation on the early life history stages of a rare cold sand-desert legume
Source: Sci Rep. 2018 Feb 19;8:3240. doi: 10.1038/s41598-018-21487-7 (PMC5818501; doi:10.1038/s41598-018-21487-7)
Supplement: Supplementary file 5 — Supplementary Table S3. [file 41598_2018_21487_MOESM5_ESM.pdf]

Effects of predispersal insect seed predation on the early life history stages of a rare cold sand-desert legume

Yi J. Han[1], Jerry M. Baskin[1, 3] , Dun Y. Tan[1,2*], Carol C. Baskin[1, 3, 4] and Ming Y. Wu[1]

[1] Xinjiang Key Laboratory of Grassland Resources and Ecology and Ministry of Education Key Laboratory for Western Arid Region Grassland Resources and Ecology, College of Grassland and Environment Sciences, Xinjiang Agricultural University, Ürümqi 830052, China

[2] College of Biology and Environmental Sciences, Jishou University, Jishou 416000, China

[3] Department of Biology, University of Kentucky, Lexington, KY 40506, USA.

[4] Department of Plant and Soil Sciences, University of Kentucky, Lexington, KY 40506, USA.

*Correspondence and requests for materials should be addressed to D.Y.T.

(tandunyan@163.com)

Supplementary Table S2. Simplified model for the effects of temperature (T), seed coat (S) (nonscarified vs. scarified), light (L) and their interaction on germination of *Astragalus lehmannianus* seeds. %SS, percentage of total sum of squares explained.

**Sources**	***d.f.***		**MS**	***F***	***P***	**SS%**
Temperature (T)	4	17591	4398	76.09	<0.001	13.23
Seed coat (S)	1	92752	92752	1604.71	<0.001	69.74
T×S	4	14269	3567	61.72	<0.001	10.73
T×S×L	4	4910	491	8.50	<0.001	3.69
