# Supplementary material for: Effects of predispersal insect seed predation on the early life history stages of a rare cold sand-desert legume
Source: Sci Rep. 2018 Feb 19;8:3240. doi: 10.1038/s41598-018-21487-7 (PMC5818501; doi:10.1038/s41598-018-21487-7)
Supplement: Supplementary file 6 — Supplementary Table S2. [file 41598_2018_21487_MOESM6_ESM.pdf]

Effects of predispersal insect seed predation on the early life history stages of a rare cold sand-desert legume

Yi J. Han[1], Jerry M. Baskin[1, 3] , Dun Y. Tan[1,2*], Carol C. Baskin[1, 3, 4] and Ming Y. Wu[1]

[1] Xinjiang Key Laboratory of Grassland Resources and Ecology and Ministry of Education Key Laboratory for Western Arid Region Grassland Resources and Ecology, College of Grassland and Environment Sciences, Xinjiang Agricultural University, Ürümqi 830052, China

[2] College of Biology and Environmental Sciences, Jishou University, Jishou 416000, China

[3] Department of Biology, University of Kentucky, Lexington, KY 40506, USA.

[4] Department of Plant and Soil Sciences, University of Kentucky, Lexington, KY 40506, USA.

*Correspondence and requests for materials should be addressed to D.Y.T.

(tandunyan@163.com)

Supplementary Table S3. Predispersal seed predation by insects in species of *Astragalus.* A dash (-) indicates no data presented.

***Astragalus* species**	**Seed predators**	**Predation (%)**	**References**
Astragalus allochrous	*Acanthoscelides mixtus*	-	1
	A. pullus	-	
A. australis	*lygaeid bug*	28.4-60.9	2
	pyralid caterpillar	28.4-60.9	
	weevil (Tychius sp.)	28.4-60.9	
A. calycosus	*Acanthoscelides aureolus*	-	1
A. canadensis	*weevil*	37.6-61	4
A. cibarius	*Acanthoscelides fraterculus*	74	5
	Bruchophagus mexicanus	25	
A.filipes	*tortricid* moth	62-93	6
	weevil	62-93	
	Acanthoscelides fraterculus	-	7
	A. pullus	-	
A. fucatus	*Acanthoscelides aureolus*	-	1
	Apanteles sp.	-	
	Bruchophagus mexicanus	-	
	Isodromus atriventris	-	
	Microdontomerrus sp	-	
A. leibergii	*Acanthoscelides fraterculus*	3-4	3,8
	Grapholita imitativa	1-8	
	Tychius semisquamosus	0	
A. lentiginosus	*Acanthoscelides aureolus*	-	9, 10
	A. mixtus	76	
	A. pullus	-	1
	Bracon bruchivorous	-	
	Bruchophagus borealis	-	

Supplementary Table S4 (continued)

	B. mexicanus	-	
	Elasmus setosiscutellatus	-	
	Horismenus productus	-	
	Liodontomerus sp.	-	
	Pteromalini sp.	-	
	Trimeromicrus maculatus	-	
	Tychius semisquamosus	-	
A. mollissimus	*Acanthoscelides aureolus*	-	1
	A. fraterculus	-	11
	A. lobatus	-	1
A. nuttalianus	*Bruchophagus borealis*	-	1
A. praelongus	*Acanthoscelides aureolus*	76	9, 10
	A. mixtus	-	
	A. pullus	-	
	Bruchophagus mexicanus	-	1
	Horismenus productus	-	
A. purshii	*Acanthoscelides fraterculus*	0.8-10	3, 8
	Grapholita imitativa	10-30	
	Tychius semisquamosus	0	
	pyralid moth	47-82	6
A. sinuatus	*Acanthoscelides fraterculus*	1-5	3, 8
	Grapholita imitativa	8-61	
	Tychius semisquamosus	0-76	
A. utahensis	*Acanthoscelides fraterculus*	60	5
A. wootoni	*Acanthoscelides aureolus*	-	1
	A. mixtus	76	9, 10
	A. pullus	-	1

1.Center, T.D. and Johnson, C.D. Host plants and parasites of some Arizona seed-feeding insects. *Ann. Entomol. Soc. Am.* **69**, 195-201 (1976).

2. Kaye, T.N. From flowering to dispersal: reproductive ecology of an endemic plant, *Astragalus australis* var. *olympicus* (Fabaceae). *Am. J. Bot.* **86**, 1248-1256 (1999).

3. Combs, J.K., Reichard, S.H., Groom, M.J., Wilderman, D.L. and Camp, P.A. Invasive competitor and native seed predators contribute to rarity of the narrow endemic *Astragalus sinuatus* Piper. *Ecol. Appl.* **21**, 2498-2509 (2011).

4. Platt, W.J. Hill, G.R. and Clark, S. Seed production in a prairie legume *(Astragalus canadensis* L.*).* Interactions between pollination, predispersal seed predation, and plant density. *Oecologia* **17**, 55-63 (1974).

5. Green, T.W. and Palmblad, I.G. Effects of insect seed predators on *Astragalus cibarius* and *Astragalus utahensis* (Leguminosae). *Ecology* **56**, 1435-1440 (1975).

6. Youtie, B.A. and Miller, R.F. Insect predation on *Astragalus filipes* and *A. purshii* seeds. *Northw. Sci.* **60**, 43-46 (1986).

7. Cane, J.H., Johnson, C., Napoles, J.R, Johnson, D.A. and Hammon, R. Seed-feeding beetles (Bruchinae, Curculionidae, Brentidae) from legumes (*Dalea ornata, Astragalus filipes*) and other forbs needed for restoring rangelands of the Intermountain West. *West. N. Am. Nat.* **73**, 477-484 (2013).

8. Combs, J.K., Lambert, A.M and Reichard, SH. Pre-dispersal seed predation is higher in a rare species than in its widespread sympatric congeners (*Astragalus*, Fabaceae). *Am. J. Bot.* **100**, 2149-2157 (2013).

9. Nelson, D.M. and Johnson, C.D. Stabilizing selection on seed size in *Astragalus* (Leguminosae) due to differential predation and differential germination. *J. Kansas Entomol. Soc.* **56**, 169-174 (1983a).

10. Nelson, D.M. and Johnson, C.D. Selenium in seeds of *Astragalus* (Leguminosae) and its effects on host preferences of bruchid beetles. *J. Kansas Entomol. Soc.* **56**, 267-272 (1983b).

11. Hamilton, J. Note on *Bruchus alboscutellatus*, *Miarus hispidulus*, *Coeliodes acephalus*, and a new *Thiobius*. *Entomol. News* **3**, 253-255 (1892).
